# Supplementary material for: Water striders adjust leg movement speed to optimize takeoff velocity for their morphology
Source: Nat Commun. 2016 Dec 7;7:13698. doi: 10.1038/ncomms13698 (PMC5150985; doi:10.1038/ncomms13698)
Supplement: Supplementary Information — Supplementary Figures 1-8, Supplementary Tables, Supplementary Notes and Supplementary References [file ncomms13698-s1.pdf]

Supplementary Figures

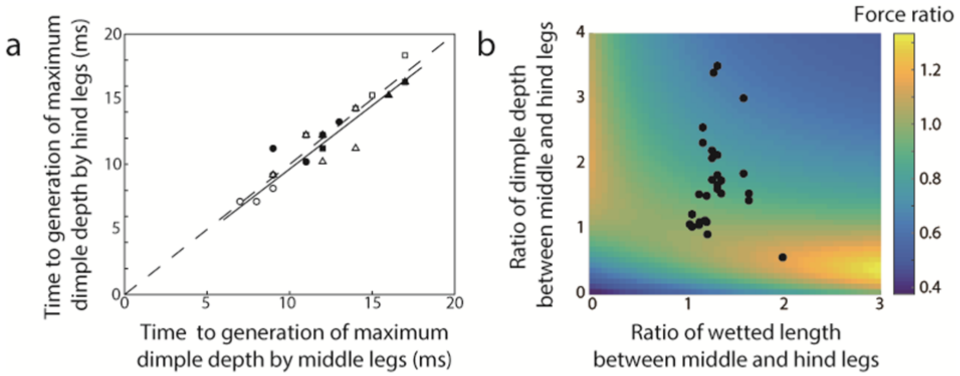

Supplementary Figure 1. Validation of synchronous motion of four legs (**a**) Comparison of the moment of maximum depth of dimple generation t_m between middle and hind legs. The correlation in each trial results in the correlation coefficient r = 0.943, p-value = 0.0311, and df = 28 implying the synchronous motion of four legs. Data from the jump of females (filled symbols) and males (unfilled symbols) of *G. remigis* (inverted triangles), *G. comatus* (diamonds), *G. latiabdominis* (circles), *G. gracilicornis* (triangles), and *A. paludum* (squares) with nymph of *G. remigis* (stars) are plotted. The dashed line indicates the exact match between middle and hind legs, and the solid line the fitted regression line. (**b**) The ratio of the force calculated with mean values of the wetted length and dimple depth of middle and hind legs to the force with different values of the wetted length and dimple depth of middle and hind legs, as a function of the ratio of wetted lengths and dimple depths made by middle and hind legs. The black dots indicate the observed jumps of water striders. The observed conditions have force ratios between 0.76 and 1.15 implying that our simplification is reasonable, except for the three cases with the highest dimple depth ratio, where the maximum dimple depths made by hind legs were below 1 mm and the resulting force ratio about 0.65. Under these conditions, the force F can be simplified in terms of C, being the mean values of the flexibility factor, l_w, the wetted length of the leg and h, the dimple depth of the four legs, with given liquid properties.

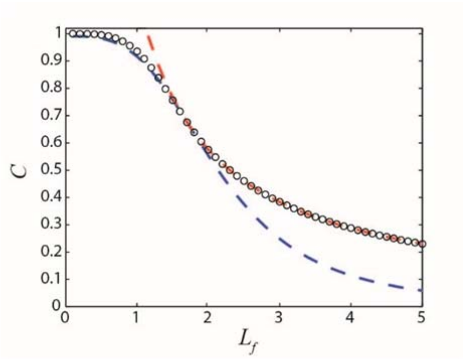

Supplementary Figure 2. Flexibility factor A flexibility factor C of a long thin flexible cylinder as a function of the scaled length L_f. Circles correspond to the numerically calculated values of C; the blue dashed line $C = (1 + 0.082L_f^{3.3})^{-1}$, and the red dashed line $C = (1.15L_f)^{-1}$. The blue dashed line is used in this study for $L_f < 2$.

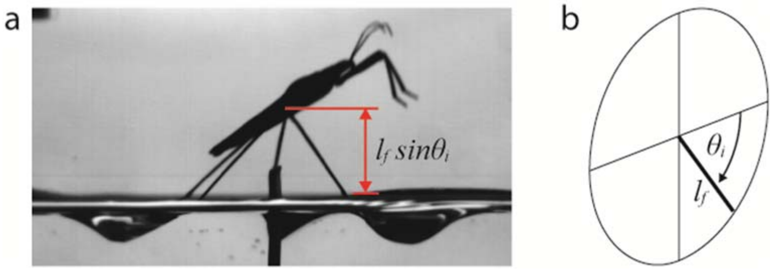

Supplementary Figure 3. Angle of rotation of a water strider's leg (**a**) The instantaneous vertical length of femur. (**b**) The angle of a leg θ_i in a plane of leg rotation with respect to the horizontal plane. The thick solid line indicates the femur, and the tired circle means the plane of leg rotation.

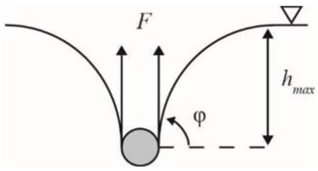

Supplementary Figure 4. Theoretical sinking depth of a cylinder The maximum deformation of the meniscus due to a thin rigid cylinder floating on a surface of the liquid, with the interfacial inclination φ and the displacement of cylinder h_{max}.

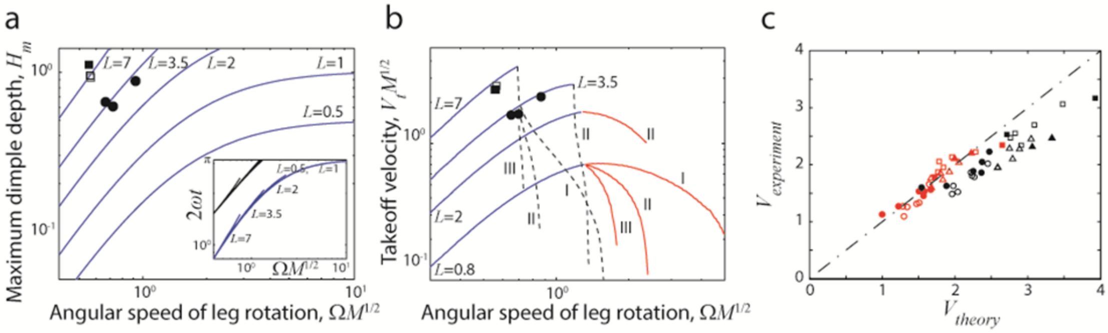

Supplementary Figure 5. The model predicts maximal dimple depth and take-off velocity

(**a**) Predicted and observed effect of the dimensionless index $\Omega M^{1/2}$, representing largely variation in leg rotation, on the dimensionless maximum dimple depth (H_m) across a range of the dimensionless maximal reach of the leg (L). (Inset: ωt versus $\Omega M^{1/2}$ at which meniscus reaches maximum depth (at $t = t_m$; blue lines), and the end of propulsion (at $t = t_e$; black lines).) (**b**) Predicted and observed effect of the dimensionless index $\Omega M^{1/2}$, representing largely variation in leg rotation, on the take-off velocity index ($V_t M^{1/2}$) for various L through the jump modes of post-takeoff closing (blue solid lines), pre-takeoff closing (red solid lines), and meniscus breaking (black dashed lines). The lines marked with roman numbers indicate the different dimensionless body mass M (I, M = 0.1; II, M = 0.5; III, M = 2.0). (**c**) Experimentally measured, dimensionless vertical velocity of water striders versus theoretical predictions at the moment of maximum dimple depth (red symbols) and takeoff (black symbols). Dashed dot line indicates the exact match between experiment and theory. In (a) and (b), the empirical values from water striders with $L \approx 3.5$ (circles; *G. latiabdominis*) and $L \approx 7$ (squares; *A. paludum*) are given. In (c), the empirical results from the jump characteristics of females (filled symbols) and males (unfilled symbols) of *G. latiabdominis* (circles), *G. gracilicornis* (triangles), and *A. paludum* (squares) are plotted. Overestimation of takeoff velocity in (c) may come from the delay of retraction of the water surface in the closing stage of real jump[1]. Dimples remaining after the legs completely take off the water surface in Fig. 1e (t = 25 ms) imply that the water surface retracts slower than the legs escaping from the water surface. Therefore, dimple depth would not reflect the exact capillary force supporting the legs but exaggerate it in the closing stage.

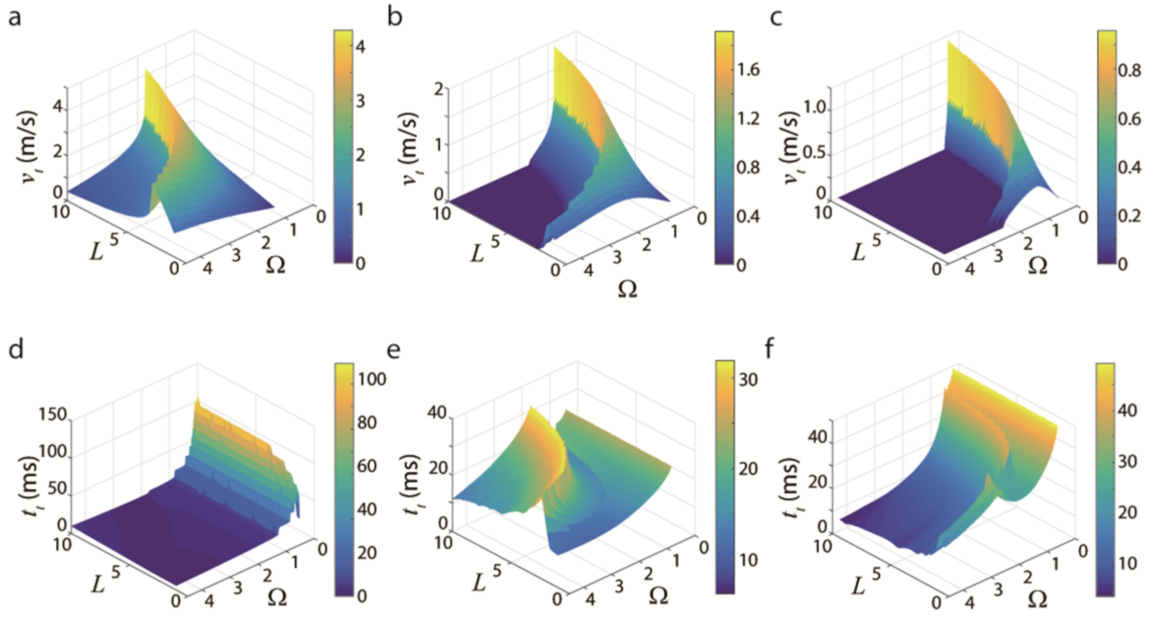

Supplementary Figure 6. Vertical takeoff velocity estimation Theoretical predictions of takeoff velocity (**a** to **c**) and the time to escape from water (**d** to **f**) with different dimensionless parameters *M*, *L*, and *Ω*. The figures are 3-dimensional representations of Fig. 4(a to f) with the same labels.

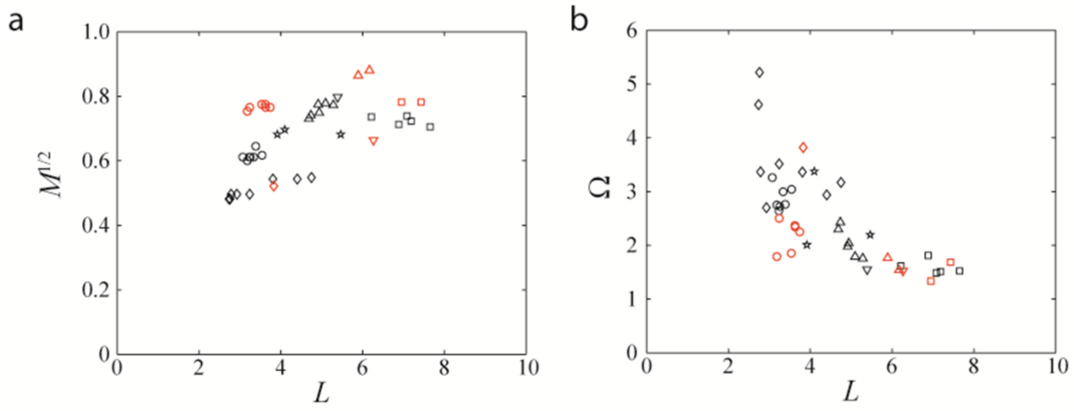

Supplementary Figure 7. Empirical values of $M^{1/2}$ and Ω The two elements of the variable $\Omega M^{1/2}$, as a function of the morphological variable *L.* (**a**) Distribution of the square root of the dimensionless body mass of water striders $M^{1/2}$ obtained from experiment with respect to dimensionless downward stroke *L*. (**b**) Distribution of dimensionless angular velocity of leg rotation of water striders obtained from experiment Ω with respect to dimensionless maximal reach of the leg *L***.** The symbols indicate jump characteristics of females (black symbols) and males (red symbols) of *G. remigis* (inverted triangles), *G. comatus* (diamonds), *G. latiabdominis* (circles), *G. gracilicornis* (triangles), and *A. paludum* (squares), and nymph of *G. remigis* (stars)**.**

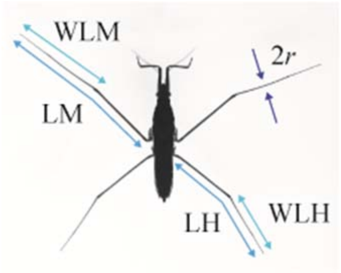

Supplementary Figure 8. Definition of lengths of legs in Supplementary Table 1

Supplementary Table

Supplementary Table 1. Body dimensions of water striders used in this study (mean ± standard deviation)

Species	Sex	No.s of jumps/ individ-uals filmed	No.s of individ-uals measur-ed	Body mass (mg)	Legnth of middle leg (mm)	Length of hind leg (mm)	Wetted length of middle leg (mm)	Wetted length of hind leg (mm)	Average radius of tibia (µm)
Symbol**					LM	LH	WLM	WLH	*r*
Gerris remigis	male	4/2 *	1*	29.3	20.0	16.6	11.4	8.6	159
	female	1/1	1	41.8	20.0	16.7	11.2	8.6	165
	nymph	3/2	2	23.2 ± 0.4	16.0 ± 0.7	12.2 ± 0.4	8.9 ± 0.5	5.8	156 ± 3
Gerris comatus	male	5/3	5	11.5 ± 2.3	14.0 ± 1.3	10.1 ± 1.3	8.0 ± 0.8	4.5 ± 0.6	96 ± 18
	female	1/1	1	10.3	12.6	9.1	7.4	4.0	88
Gerris latiabdominis	male	7/4	4	14.7 ± 0.4	12.5 ± 0.2	9.3 ± 0.2	7.2 ± 0.2	4.4 ± 0.2	89 ± 2
	female	6/3	3	24.3 ± 1.2	13.3 ± 0.2	10.2 ± 0.2	7.6 ± 0.1	4.9 ± 0.2	99 ± 2
Gerris gracilicornis	male	6/6	6	29.0 ± 2.5	18.3 ± 0.7	13.3 ± 0.5	9.9 ± 0.5	5.4 ± 0.5	131 ± 7
	female	2/2	2	48.5 ± 2.7	21.0 ± 0.4	16.5 ± 0.1	11.4 ± 0.5	7.7 ± 0.2	143 ± 3
Aquarius paludum	male	5/5	5	37.7 ± 0.9	24.0 ± 1.0	21.0 ± 1.2	12.7 ± 0.5	8.9 ± 0.7	130 ± 5
	female	2/1	1	49.0	24.4	21.4	13.2	9.1	142

* In the case of one individual *G. remigis* male, we did not collect measurements because it escaped during filming. In calculations for this individual *G. remigis* male we used the measurements collected from another male, who was similar in size and morphology (was also filmed). For all remaining species/sexes we measured every individual that was filmed (for some species we measured more individuals).

** Corresponding symbols in Supplementary Fig. 8.

Supplementary Notes

Supplementary Note 1. Verification of the assumption of four legs moving synchronously

To model the vertical velocity of a water strider's centre of mass, the forces acting on its four legs were added. In the model, we assumed that all the legs involved in the propulsion move synchronously and leave the surface at the same time. This assumption is verified by correlation analysis between the moments the maximum dimple depth of middle and hind legs are reached in each trial, resulting in the correlation coefficient r = 0.943, p-value = 0.0311, and df = 28 (Supplementary Fig. 1a).

In addition, we used average values of wetted length and resulting dimple depth made by middle and hind legs. We exploit this simplification because equations of motion become tractable and the corresponding theoretical predictions are accurate enough. Supplementary Fig. 1b shows the verification of this simplification. The color map indicates the ratio of two forces (see Supplementary Note 2) fourfold of the force $\bar{F}$ calculated with mean values of the wetted length $\bar{l}_w$ and dimple depth $\bar{h}$ of middle and hind legs to the sum of the forces on the four legs with different values of the wetted length and dimple depth of middle and hind legs:

$$\frac{4\bar{F}}{\sum F} = \frac{4\bar{l}_w\bar{h}\left[1-(\bar{h}/2l_c)^2\right]^{1/2}}{\sum l_w h\left[1-(h/2l_c)^2\right]^{1/2}}. \qquad (1)$$

The black dots show the measured value from jumping of water striders we observed when the legs reach the deepest position. The observed conditions have force ratios between 0.76 and 1.15 implying that our simplification is reasonable, except for the three cases with the highest dimple depth ratio, where the maximum dimple depths made by hind legs were below 1 mm and the resulting force ratio about 0.65. Under these conditions, the force F can be simplified in terms of C, being the mean values of the flexibility factor, l_w, the wetted length of the leg and h, the dimple depth of the four legs, with given liquid properties.

Supplementary Note 2. Capillary force on a leg

Since water strider legs bend during a jump, the flexibility of the cylinder needs to be taken into account in modeling the force exerted on the legs. Vella[2] provided the numerical solutions of capillary force acting on a long thin flexible cylinder clamped horizontally at one end and held at a given depth under the free surface. According to the study, the capillary force on a rigid thin cylinder can be written as

$$F_r = 2\rho g l_c l_w h\{1 - [h/(2l_c)]^2\}^{1/2}, \qquad (2)$$

where l_w denotes the wetted length of the cylinder. F_r monotonically increases with the depth of dimple h while $h < \sqrt{2}l_c$. For a flexible cylinder, the scaled length $L_f = l_w/l_{ec}$ plays an important role, where $l_{ec} = (Bl_c/\sigma)^{1/4}$ is the modified elasto-capillary length of the cylinder with bending rigidity $B = \pi E r^4/4$. Here E corresponds to Young's modulus of insect's cuticle and r is the radius of leg. Vella presented the numerical solutions of supporting force on bent cylinders with various L_f revealing that flexibility hardly changes the shapes of the force curves with different depth, but decreases the magnitude of the force quantitatively. In other words, more flexible cylinders having larger L_f generate weaker supporting forces.

To transform the numerical solutions into more practical forms, here we suggest an approximate force equation by introducing a flexibility factor C of the cylinder as a function of L_f. Then the capillary force on a flexible cylinder is simply estimated as

$$F = 2\rho g l_c C l_w h\{1 - [h/(2l_c)]^2\}^{1/2}. \qquad (3)$$

As a result, the effective wetted length becomes Cl_w because it replaces l_w in the formula of F_r for a rigid cylinder. C of each L_f was calculated by averaging the ratios of the numerical solution of the capillary force on a flexible cylinder to the asymptotic solution of that on a rigid cylinder as the dimple depth h varies from 0 to $\sqrt{2}l_c$. Given standard liquid properties and gravitational acceleration, we simplify C into a function of L_f using the curve fit $C \approx (1 + 0.082L_f^{3.3})^{-1}$ for $L_f < 2$ or $C \approx (1.15L_f)^{-1}$ for $L_f > 2$ (Supplementary Fig. 2). The factor C decreases with L_f, implying weaker capillary force on the more flexible cylinder. To calculate flexibility factor of water striders, we used the relationship $C \approx (1 + 0.082L_f^{3.3})^{-1}$ as indicated by blue dashed line in Supplementary Fig. 2, since all the water striders tested have the scaled length L_f shorter than 1.5.

Supplementary Note 3. The measurement of rotation angle

The angle of legs θ was calculated by averaging the angle of each leg θ_i with respect to the horizontal plane of a water strider from the video. The angle of each leg θ_i was obtained by measuring the instantaneous vertical length of femur, $l_f \sin\theta_i$, with given length of femur l_f, as shown in Supplementary Fig. 3.

Supplementary Note 4. The critical depth of meniscus breaking

We observed several cases in which a leg quickly sank under the water surface after the distal end of the leg pierced the meniscus during the stroke. In these cases, the capillary force on the leg could be neglected upon penetration of meniscus because of the rapid decrease of the wetted length. This water surface piercing can be predicted from the theoretical calculations for rigid cylinders[2-4]: the maximum displacement of the centre of a thin rigid cylinder at the gas-liquid interface before sinking is modeled to be reached at an interfacial inclination φ of $\pi/2$ and the displacement of cylinder (h_{max}) of $\sqrt{2}l_c$, as illustrated in Supplementary Fig. 4. The average depth reached by the distal end of the legs and by the lowest parts of the legs upon the surface penetration (corresponding to the depth of dimple at the moment of penetration) were 3.72 and 4.40 mm, respectively. Both the values are comparable to the maximum theoretical depth of a floating rigid cylinder ($\sqrt{2}l_c$, 3.84 mm for water). Therefore, in the model, we take $\sqrt{2}l_c$ as the critical depth h_{max} under which the surface penetration would occur. In addition, we note that the maximum depth limit is equivalent to the maximum force limit[1], or the force per unit wetted length f should satisfy $f < 2\sigma$, because capillary force on a leg is determined by the dimple depth[2, 3].

Supplementary Note 5. The model predicts maximal dimple depth observed in insects

We solved equation (3) in the main text and plotted the theoretically predicted maximum dimple depth as a function of the dimensionless maximal reach of the leg L (femur+tibia+tarsus) and dimensionless index combining angular velocity of leg rotation,

body mass and tibia plus tarsus length $\Omega M^{1/2}$ in Supplementary Fig. 5a, which reflects morphological and behavioural trait, respectively. Strictly speaking, $\Omega M^{1/2}$ is a function of behaviour ($\Omega = \omega(l_c/g)^{1/2}$) and morphology (a function of body mass and the length of tibia+tarsus; $M = m/\rho l_c^2 C l_t$). But, for a given species-specific morphology (M) the variation in $\Omega M^{1/2}$ represents behavioural variation in angular velocity of the legs. Additionally, for among-species comparisons, a unit change in morphology affects $\Omega M^{1/2}$ less than a unit change in Ω does, justifying our approximate view of $\Omega M^{1/2}$ as largely a behavioural index (See Supplementary Note 8, and Supplementary Fig. 7 for more explanations).

The maximum dimple depth increases with the increasing $\Omega M^{1/2}$ or with the increasing L, for an individual water strider with given m, l_t, and C, and then it tends to converge to L. This asymptotic maximum dimple depth corresponds to the stroke with extremely high speed without any upward displacement of the body. However, the dimple depth H can grow only until the meniscus breaks[3, 4] (see Supplementary Fig. 4 and Supplementary Note 4). The predictions match empirical results, as exemplified in Supplementary Fig. 5a for two water strider species (*G. latiabdominis* and *A. paludum*).

Supplementary Note 6. The model predicts take off velocity observed in insects

Takeoff velocity of a water strider is obtained via integrating the instantaneous net force on the body, which depends on the dimple depth, over time until the end of legs reach the zero depth position ($t = t_t$). Supplementary Fig. 5b presents the predicted dimensionless takeoff velocity $V_t = v_t (gl_c)^{-1/2}$ multiplied by $M^{1/2}$ with different $\Omega M^{1/2}$ and L. As the water strider's stroke with given morphology becomes gradually faster, the mode of jump switches from post-takeoff closing jump to pre-takeoff closing or meniscus breaking jump depending on the maximal reach of the leg L. For the long maximal reach ($L > \sqrt{2}$), the takeoff velocity sharply drops as $\Omega M^{1/2}$ exceeds a certain critical value because of the rupture of meniscus. For pre-takeoff closing jump or meniscus breaking jump, V_t varies with M because the insect would go into a free fall after closing of legs or meniscus breaking. Meniscus breaking jump is less beneficial because the support from the water surface is not strong in the late stage of jump. This may cause not only the drag when the submerged legs rise but also destabilization of the takeoff trajectory by various disturbances, such as wind gusts or other environmental effects, to which small animals like water striders may be susceptible. Moreover, during the time between the instant of meniscus breaking t_b or the end of closing of the legs t_c and the instant of takeoff t_t of meniscus breaking jump or pre-takeoff close jump, the insect is almost in a free fall resulting in the decrease in takeoff velocity ($V_t M^{1/2} = [V(t_b)^2 M - 2H(t_b)M]^{1/2}$ or $[V(t_c)^2 M - 2H(t_c)M)]^{1/2}$) because of a lack of supporting force. We have verified that the theoretical predictions of takeoff velocity calculated with the measured L and $\Omega M^{1/2}$ agree reasonably well with the experimental measurements on five species of water striders (see Supplementary Fig. 5c).

Supplementary Note 7. Three dimensional graphs of theoretical results of takeoff velocity and latency

Supplementary Fig. 6 shows the three dimensional graphical representation of Fig. 4a to f. In Supplementary Fig. 6a-c, the 3D versions of these prediction for maximal speed effectively show the dramatic decrease in performance after the surface breaking threshold is reached. In Supplementary Fig. 6d to f, the 3D versions of these predictions effectively show a very narrow range of low t_t in the area just below the meniscus-breaking threshold.

Supplementary Note 8. Variation in $\Omega M^{1/2}$ as an index of variation in the leg rotation velocity

In this study, there are three important parameters to explain the water striders' jumping performance on water, dimensionless angular speed of leg rotation $\Omega = \omega(l_c/g)^{1/2}$, dimensionless body mass $M = m/(\rho l_c^2 C l_t)$, and dimensionless maximal reach of the leg $L = \Delta l/l_c$. However, in the final model predictions (Fig. 4g of the main text) the results are presented in the two dimensional space of $\Omega M^{1/2}$ and L. The values of water striders' dimensionless angular velocity of leg rotation, Ω, extracted from the videos varied within an approximate range of [1.2–5.5], while dimensionless body mass M varied only within an approximate range of [0.25–0.85]. But, the square root of dimensionless body mass, $M^{1/2}$, varied even less (Supplementary Fig. 7). Therefore, variation in $\Omega M^{1/2}$ can be treated as an indicator of variation in the leg rotation Ω rather than mass M. Additionally, it seems that water striders with longer dimensionless maximal reach of the leg L used slower leg rotation Ω, (Supplementary Fig. 7b), and that the analogical association between L and $M^{1/2}$ (Supplementary Fig. 7a) was not as clear as between L and Ω.

Supplementary Note 9. Simplified relation between L and $\Omega M^{1/2}$

Equation (2) in the main text can be rewritten as $v = v_s$ at $t = t_m$, where $v = \frac{1}{m}\int_0^{t_m} F\mathrm{d}t$, $F = 8\rho g l_c C l_w h\{1 - [h/(2l_c)]^2\}^{1/2}$ and $v_s = \omega \Delta l \sin(2\omega t)$, because when the legs reach the deepest position, the rate of dimple growth dh/dt becomes zero. With rough approximations of $h \sim Ut$, $U \sim h_m/t_m$, $h_m \sim l_c$, and $F \sim \rho g l_c C l_t h$, v at $t = t_m$ can be simplified to $v \sim \frac{\rho g l_c C l_t}{\omega m}\int_0^{\omega t_m} \frac{l_c \omega t}{\omega t_m}\mathrm{d}\omega t \sim \rho g l_c^2 C l_t \omega t_m/(\omega m)$. Then, by balancing this relation with v_s, we can get the relation $\Delta l \sim (\rho g l_c^2 C l_t \omega t_m)/[\omega^2 m \sin(2\omega t_m)]$, which can be further simplified to $\Delta l \sim (\rho g l_c^3 C l_t)^{1/2}/(\omega m^{1/2})$ by substituting $\omega t_m \sim \Omega M^{1/2}$ and $\sin(2\omega t_m) \sim 1$ (see the inset of Supplementary Fig. 5a). Thus, we get $L \sim \Omega^{-1} M^{-1/2}$.

Supplementary References

1. Koh, J.-S. *et al*. Jumping on water: Surface tension-dominated jumping of water striders and robotic insects. *Science* **349**, 517-521 (2015).
2. Vella, D. Floating objects with finite resistance to bending. *Langmuir* **24**, 8701-8706 (2008).
3. Vella, D., Lee, D. G. & Kim, H.-Y. The load supported by small floating objects. *Langmuir* **22**, 5979-5981 (2006).
4. Shi, F. *et al.* Towards understanding why a superhydrophobic coating is needed by water striders. *Adv. Mater.* **19**, 2257-2261 (2007).
